# Antibiotic Treatment for Chronic Rhinosinusitis: Prescription Patterns and Associations With Patient Outcome and the Sinus Microbiota

**DOI:** 10.3389/fmicb.2020.595555

**Published:** 2020-12-22

**Authors:** Christian A. Lux, Brett Wagner Mackenzie, James Johnston, Melissa Zoing, Kristi Biswas, Michael W. Taylor, Richard G. Douglas

**Affiliations:** ^1^School of Biological Sciences, University of Auckland, Auckland, New Zealand; ^2^Department of Surgery, School of Medicine, University of Auckland, Auckland, New Zealand

**Keywords:** chronic rhinosinusitis, sinus microbiota, clinical microbiology, antibiotic therapy, antibiotic policy

## Abstract

**Background:**

Chronic rhinosinusitis (CRS) is a common and debilitating inflammatory condition of the sinuses, afflicting 5% of the general population. Although antibiotics are frequently prescribed for the medical management of CRS, there is surprisingly little evidence to support their efficacy. In this study, we aimed to establish associations between medication usage, the sinus microbiota and patients’ clinical outcomes.

**Methods:**

Antibiotic prescription patterns for the year before sample collection of 156 CRS patients, 45 disease control patients (mostly requiring septoplasty and inferior turbinate reduction) and 35 healthy control subjects were examined and analyzed together with previously published bacterial 16S rRNA gene amplicon data from our group.

**Results:**

The highest antibiotic usage was observed among the two CRS patient categories. Despite heavy antibiotic usage, CRS patients’ clinical outcomes as indicated by patient questionnaires and radiologic scores were similar to those patients that did not receive any antibiotics. The sinus microbiota was dominated by members of the bacterial genera *Corynebacterium* and *Staphylococcus* in all three cohorts. Bacterial community dispersion as measured by principal coordinate analysis was significantly higher in CRS patients compared to healthy control subjects, but not disease control patients. Pairwise comparisons within cohorts revealed differences in the relative 16S rRNA gene sequence abundances of the genera *Staphylococcus* and *Lawsonella* between antibiotic users and non-users. However, overall antibiotic effects were minimal and unpredictable.

**Conclusion:**

The unpredictable effects of antibiotic treatment on the sinus microbiota found in this study, together with the lack of differences in patients’ symptom scores between cohorts, do not support preoperative antibiotic treatment for CRS patients.

## Introduction

Chronic rhinosinusitis (CRS) is a common and debilitating condition, afflicting about 5% of the general population (Dietz de Loos et al., 2019; Hwang et al., 2019). CRS is initially treated with a combination of saline rinses, corticosteroids and antibiotics (Rosenfeld et al., 2015; Malaty, 2016; Chandy et al., 2019). In 2013, sinusitis was the diagnosis which accounted for the most outpatient prescriptions for antibiotics in the US, and CRS was responsible for two-thirds of the prescriptions for this diagnosis (Smith et al., 2013). The economic burden of CRS is significant, with antibiotics accounting for approximately 60% of medical treatment costs (Beule, 2015).

Although antibiotics are frequently prescribed for the medical management of CRS, there is surprisingly little evidence to support their efficacy (Lim et al., 2008; Head et al., 2016). Three studies so far have found a moderate improvement in symptoms of CRS with and without nasal polyps after receiving macrolide antibiotics for between three and six months (Wallwork et al., 2006; Zeng et al., 2011; Varvyanskaya and Lopatin, 2014). However, this observed improvement may well be due to the anti-inflammatory properties of macrolides rather than their antimicrobial activity (Hoggard et al., 2017b; Walker et al., 2019). Because of the paucity of evidence supporting their efficacy for treatment of CRS, antibiotic use is not routinely recommended in several clinical guidelines (Fokkens et al., 2012; Cain and Lal, 2013; Kennedy and Borish, 2013; Bachert et al., 2014; Orlandi et al., 2016; Barshak and Durand, 2017). Nonetheless, the administration of antibiotics remains widespread practice in the management of CRS (Akkerman et al., 2005; Gulliford et al., 2014). Of those patients who progress to surgery, almost all will have been prescribed multiple courses of antibiotics and corticosteroids (Dubin et al., 2007; Schwartz et al., 2016) and postoperatively, medical treatment is routinely continued (Wood et al., 2017).

Side-effects are commonly associated with antibiotics, particularly when they are taken for longer periods (Walker et al., 2019). There are additional concerns regarding the over-use of antibiotics and an associated increase in antibiotic resistance, which represents a considerable threat to the efficacy of these drugs (Lodato and Kaplan, 2013). In CRS patients, resistance against second generation cephalosporins for *Haemophilus influenzae* and *Moraxella catarrhalis* has been noted (Brook, 2017). Additionally, resistance against penicillins (e.g., amoxicillin) and sulfonamides (co-trimoxazole) in bacterial isolates from CRS patients has been recently reported (Musa et al., 2019).

Inappropriate antibiotic treatment may alter the structure of the natural commensal bacterial community. Antibiotics may have a detrimental effect on commensal organisms and thereby destabilize the indigenous microbiota. Lacking the commensals’ protective effects can result in an increasing susceptibility to colonization by pathogenic bacteria (Khan et al., 2019). Antibiotic treatment has been associated with disease exacerbation in an animal model of CRS as well as in patients with cystic fibrosis (Abreu et al., 2012; Lim et al., 2014). Destabilization of the bacterial community and reduced microbial diversity, as a result of antibiotic treatment, may predispose patients to secondary infection and development of chronic inflammation (Fodor et al., 2012; Zhao et al., 2012). In one study, antibiotic treatment for any other indication was associated with an increased risk of developing CRS (Maxfield et al., 2017).

In this study we examined antibiotic prescription patterns throughout the year prior to sample collection for CRS patients, disease control patients, and healthy control subjects in New Zealand. We correlated these and other clinical data (including age, gender, and co-morbidities) with previously published bacterial amplicon sequencing data to establish associations between medication usage, sinus bacterial community composition and CRS patients’ clinical outcomes.

## Materials and Methods

### Cohorts and Data Acquisition

Patient clinical and bacterial 16S rRNA gene amplicon sequencing data were obtained from previous studies conducted by our research group (Biswas et al., 2017, 2019; Hoggard et al., 2017a; Jain et al., 2018; Wagner Mackenzie et al., 2019a; Lux et al., 2020). Amplicon sequence data originating from intra-operatively collected swab or tissue samples from 156 CRS patients diagnosed according to the EPOS 2012 criteria (Fokkens et al., 2012) and 45 disease control patients collected between 2015 and 2018 were collated (Supplementary Table 1). CRS patients were further delineated based on the presence of nasal polyps (CRSsNP = CRS without nasal polyps, CRSwNP = CRS with nasal polyps). Disease controls refer to patients that underwent sinus surgery for reasons unrelated to sinusitis, such as septoplasty and turbinate reduction, removal of pituitary adenomas or medial orbital decompression for Grave’s ophthalmopathy. Disease control patients with a Lund-Mackay score >4 were excluded to ensure inclusion of patients with minor sinus opacification (not indicative of sinusitis) only. An additional 35 healthy subjects who were asymptomatic, had no history of sinus surgery and no antibiotic usage during 4 weeks prior to sample collection were also included in this study. Healthy volunteers were recruited at the Auckland hospital and medical school and middle meatus swab samples were collected under endoscopic guidance. Data on antibiotic intake were collected as yes/no answers to the question if subjects were prescribed any antibiotics during the past 12 months. Nucleic acids were extracted and 16S rRNA genes amplified for sequencing as previously described (Hoggard et al., 2017a). Raw sequences are publicly available on the NCBI website with accession number PRJNA639382.

Bacterial community composition data were determined from paired swabs that were collected from the left middle meatus under endoscopic guidance, with the exception of samples from one study (*n* = 10) in which DNA was extracted from bulla ethmoidalis tissue biopsies (Biswas et al., 2019). We have previously shown a high correlation between the microbiota determined by middle meatal swabs and tissue from the bulla ethmoidalis (Kim et al., 2015).

Extensive clinical data, including medical prescriptions for the 12 months preceding sample collection, were gathered for patients from the CRS and disease control groups from the hospital’s electronic patient record system (*Concerto*, Leicester, United Kingdom) used in New Zealand. Every prescription for antibiotics throughout the year prior to sample collection was analyzed. The type of antibiotic, number of courses and (due to the lack of information on usage days and dosage) the total number of prescribed tablets were recorded. Data were excluded if the number of antibiotic tablets was not included in the prescription information. Other clinical data including age, gender, smoking status and co-morbidities were recorded. Lund-Mackay scores and 22-item sinonasal outcome test (SNOT-22) scores, both recorded at the last clinical visit before surgery, were also documented as measures for patient outcome. Informed, written consent was given by each patient and extraction of clinical data from the Concerto database was approved by the New Zealand Health and Disability Ethics Committee (NTX/08/12/126).

### Sequence Processing and Quality Control

All studies included in our analyses targeted the V3–V4 hypervariable regions of the bacterial 16S RNA gene using primers 341F 5′-CCTACGGGNGGCWGCAG-3′ and 785R 5′-GACTACHVGGGTATCTAATCC-3′ (Klindworth et al., 2013). Amplicon sequence data were generated on the Illumina MiSeq platform with 2 × 300 base-pairs (bp), paired-end sequencing except for a subset of data from one study (Hoggard et al., 2017a) in which a 2 × 250 bp, paired-end sequencing approach was used. All raw data were re-analyzed following the DADA2 pipeline (Callahan et al., 2016). The DADA2 software package (“dada2” v1.12.1) was employed as implemented in R (v3.6.1) (R Core Team, 2019). Briefly, raw sequencing reads were quality filtered and dereplicated before denoising was performed based on previously calculated, dataset-specific error models. Amplicon sequence variant (ASV) inference was performed using the DADA2 algorithm with pseudo (approximated) pooled processing for increased sensitivity to sequence variants that may be present at very low frequencies in multiple samples. Forward and reverse sequence pairs were merged and an ASV table was constructed. Sequences much shorter than or substantially exceeding the expected insert size (i.e., sequences <380 and >450 bp) were removed and an additional filtering step was performed to remove chimeras. Taxonomic assignment of each ASV was performed using the naïve Bayesian classifier implementation in the DADA2 package with the SILVA reference dataset (v128) (Quast et al., 2013). ASVs classified as eukaryotic or chloroplast were removed and the ASV table rarefied to 2000 reads per sample.

### Statistical Analysis of Demographic and Clinical Data

Analysis of combined clinical and microbiota data was conducted in the R software environment (v3.6.1). Statistical comparisons between subgroups of CRS patients, disease control patients and healthy control subjects were conducted. Differences were regarded as significant based on three levels, namely *p* < 0.05 (^∗^), *p* < 0.01 (^∗∗^), and *p* < 0.001 (^∗∗∗^).

Overall differences between groups were tested using *Chi*-square test or Fisher’s exact test (for groups with *n* < 5) for categorical variables. Analysis of variance (ANOVA) or Kruskal-Wallis test (for non-normally distributed data) was used for comparisons of continuous variables. Categorical variables of interest with significant differences were further examined by pairwise *Chi*-square or Fisher tests with the Benjamini-Hochberg adjustment for multiple comparisons using the “pairwiseNominalIndependence” command from the rcompanion package (v2.3.7) (Mangiafico, 2019). For differences between continuous variables, Tukey’s HSD and Dunn’s test were performed following ANOVA and Kruskal-Wallis test, respectively. To investigate associations between antibiotic treatment and symptom scores, a Spearman correlation test was performed using the “cor.test” command. The Pearson Product-Moment correlation was used to analyze the association between single ASVs and bacterial community dispersion.

### Bacterial Community Data

The “estimate_richness” function of the phyloseq package (v1.28.0) (McMurdie and Holmes, 2013) was applied to calculate alpha diversity according to observed richness, Shannon and inverse Simpson metrics. Pairwise comparisons between groups were conducted using the Wilcoxon test for each alpha diversity metric. Beta diversity was analyzed using a Bray-Curtis dissimilarity matrix generated with the “vegdist” command from the vegan package (v2.5–6) (Oksanen et al., 2019). To visualize differences between subgroups, a principal coordinate analysis (PCoA) was performed on this data with the “cmdscale” command. To determine if different cohort-specific variables affected the structure of the sinus microbiota, we conducted a permutational multivariate analysis of variance (PERMANOVA) based on Bray-Curtis distance matrices using the “adonis” function as implemented within the vegan package with 999 permutations of data. PERMANOVA was also performed based on Bray-Curtis distance matrices to assess whether technical variations between studies (i.e., generated sequence read length and sample type) accounted for any observed variation in bacterial community structure. Pairwise comparisons of single ASV abundances were conducted using Kruskal-Wallis and *post hoc* Dunn’s test with Benjamini-Hochberg adjustment. Linear discriminant analysis (LDA) of effect size (LEfSe) was conducted to determine potential ASVs that differentiate CRS, disease control and healthy control groups. LEfSe was used as implemented within the Galaxy web application with α = 0.05 for both Kruskal-Wallis and Wilcoxon test (Segata et al., 2011). A logarithmic LDA score threshold of 3.0 for discriminative features and a multiclass analyses strategy of all-against-all was applied to determine potential markers for each cohort.

## Results

A total of 236 subjects (77 CRSsNP, 79 CRSwNP, 45 disease control, and 35 healthy control) for whom both amplicon sequencing and clinical data were available were included in this study. De-noising and quality filtering of sequence data yielded a total of 11,086,887 merged reads. After data filtering and rarefaction, 1,355 ASVs across 229 samples were retained. Additional filtering based on a minimum relative abundance of 0.01% yielded a dataset with 440 ASVs which was used for all subsequent analyses.

Healthy control subjects were significantly younger and more ethnically diverse than patients from all other groups (Table 1 and Supplementary Table 2). The CRSwNP group included significantly fewer males than the disease control group, and CRSwNP patients had significantly higher incidences of co-morbid asthma than all other groups.

**TABLE 1:** Cohort demographics and clinical information.

**Variable^*a*^**	**CRSsNP^*b*^ (*n* = 77)**	**CRSwNP^*b*^ (*n* = 79)**	**Disease control^*b*^ (*n* = 45)**	**Healthy control^*b*^ (*n* = 35)**	**Unadjusted *p*-value^*c*^**
Age	46 (19 to 77)	46 (18 to 75)	45 (19 to 82)	24 (20 to 55)	<0.001
Gender	38 of 77 (49%)	53 of 79 (67%)	29 of 45 (64%)	16 of 35 (45%)	0.007
European	69 of 77 (90%)	65 of 79 (82%)	37 of 45 (82%)	20 of 35 (57%)	<0.001
Smoker	2 of 73 (3%)	7 of 77 (9%)	4 of 45 (9%)	0 of 35 (0%)	0.011
Asthma	30 of 69 (43%)	42 of 72 (65%)	6 of 43 (14%)	NA	<0.001
**Medication information**					
Antibiotic usage (1 year prior to sample collection)	61 of 76 (80%)	52 of 74 (70%)	38 of 45 (84%)	8 of 35 (23%)	**<0.001**
Antibiotic usage (1 month prior to sample collection)	4 of 77 (5%)	11 of 78 (13%)	0 of 45 (0%)	0 of 35 (0%)	**0.009**
Number of antibiotics (tablets)	57 (0 to 654)	26 (0 to 397)	48 (0 to 189)	NA	**0.03**
Number of antibiotic courses	1 (0 to 12)	1 (0 to 8)	2 (0 to 4)	NA	0.178
Heavy antibiotic users^*d*^	13 of 72 (18%)	5 of 69 (7.2%)	2 of 39 (5%)	0 of 35 (0%)	**<0.001**
**Patient outcomes**					
Lund-Mackay score	***n* = 70** 13 (5 to 22)	***n* = 72** 19 (6 to 24)	***n* = 26** 0 (0 to 4)	NA	**<0.001**
SNOT-22 score	***n* = 35** 39 (4 to 85)	***n* = 33** 36 (3 to 94)	***n* = 14** 14 (0 to 57)	NA	**<0.001**

*^*a*^Categorical variables are summarized as proportion yes/total (%), except for gender, which is given as proportion female. Continuous variables are summarized as median (range).*

*^*b*^Total cohort numbers for each group are given. Differences in total numbers for each variable reflect missing data from some subjects.*

*^*c*^Overall differences between groups were tested using *Chi*-squared test or Fisher’s exact test (for groups with *n* < 5) for categorical variables and ANOVA or Kruskal-Wallis test (for non-normally distributed data) for continuous variables. Significant p-values of interest (*p* < 0.05) are noted in bold.*

*^*d*^Heavy antibiotic users are subjects that received more than 150 tablets throughout the one-year period. SNOT-22, 22 item sinonasal outcome test; CRSsNP, CRS without nasal polyps; CRSwNP, CRS with nasal polyps; NA, not available.*

### Antibiotic Prescription Patterns and Patient Outcome

**FIGURE 1:**
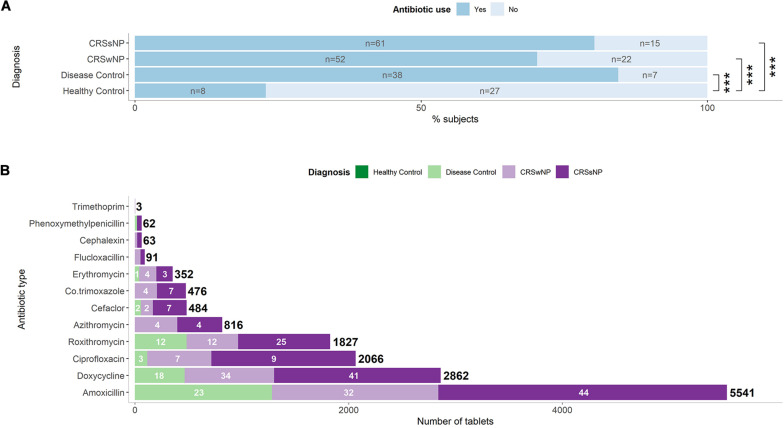
Antibiotic prescription patterns in CRS and control subjects. **(A)** Proportion of subjects that received antibiotics within one year prior to surgery. Number of patients per group are shown inside the bars. Significant differences are indicated by asterisks. **(B)** Number of tablets prescribed of each antibiotic type per group. The total numbers of tablets of each antibiotic type are indicated in bold. Numbers of subjects per group receiving a specific antibiotic are indicated in white for types with four or more users. Amoxicillin was typically prescribed in combination with clavulanic acid. Information on the number of tablets was not available for subjects from the healthy control group.

Twenty-three percent of healthy subjects took at least one course of antibiotics during the 12 months preceding sample collection (Table 1). The great majority of the disease control group (84%) took antibiotics over the same time period. The CRSsNP (80%) and CRSwNP (70%) group had similar proportions of antibiotic users (Figure 1A). In addition to examining the period 12 months preceding sample collection, we assessed antibiotic prescriptions during the 4 weeks prior to sampling to determine immediate effects of antibiotic treatment. All patients from the disease control group received no antibiotics during the 4 weeks prior to sample collection, while 5 and 13% of the CRSsNP and CRSwNP group had antibiotics during that time, respectively.

Amoxicillin-clavulanate and doxycyline were the commonly prescribed antibiotics (Figure 1B). Overall, heavy usage of antibiotics was observed in CRS and the disease control subjects. CRSsNP patients received 7,777 tablets in the year prior to surgery, which was significantly higher than the CRSwNP patients (4,594, *p* < 0.001), reflecting in part the greater number of antibiotic users among the CRSsNP cohort.

**FIGURE 2:**
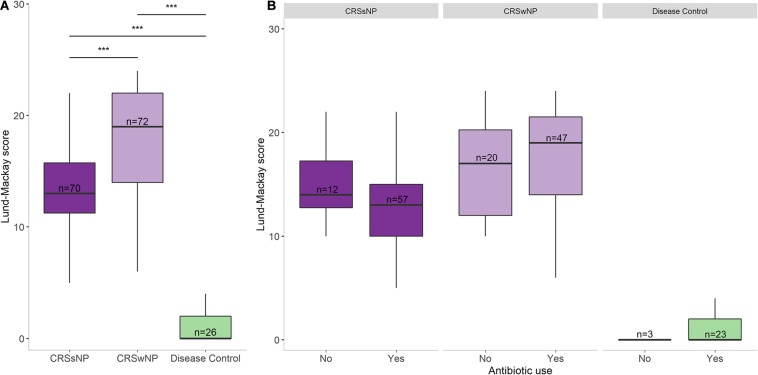
Disease severity scores and antibiotic usage. **(A)** Lund-Mackay scores for each group. **(B)** Lund-Mackay scores for each group separated into antibiotic users and non-users. Bottom and top margin of each box indicate the 25th and 75th percentile with the midline representing the median. Differences in total numbers after separating groups based on antibiotic usage reflect missing prescription data from some subjects. Statistical differences are indicated according to the following significance levels: ***p < 0.001.

SNOT-22 and Lund-Mackay scores were higher in the CRS groups than in the disease control group (*p* < 0.001, Table 1). No significant differences were observed in patient symptom scores between CRS cohorts that received antibiotic treatment in the year prior to sample collection and those that did not (Figure 2). Furthermore, there was no association between amount of antibiotics received and changes in SNOT-22 or Lund-Mackay scores.

### The Sinus Microbiota in CRS and Control Subjects

**FIGURE 3:**
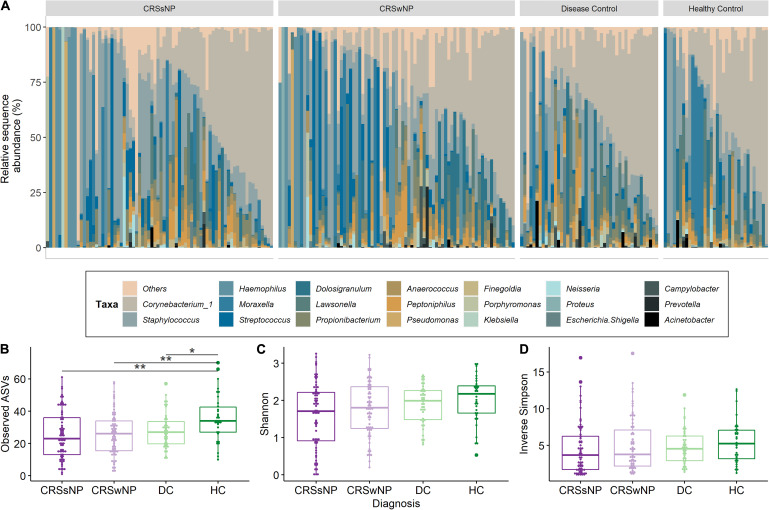
Bacterial community composition and alpha diversity for CRS and control groups. A taxa plot with the 20 most abundant genus level-assigned taxa **(A)** and alpha diversity measures for community richness (Observed ASVs; **B**), Shannon diversity **(C)** and inverse Simpson diversity **(D)** are shown. Samples in the taxa plot are arranged by decreasing relative abundance of Corynebacterium_1. Statistical differences are indicated according to the following significance levels: *p < 0.05, **p < 0.01.

**FIGURE 4:**
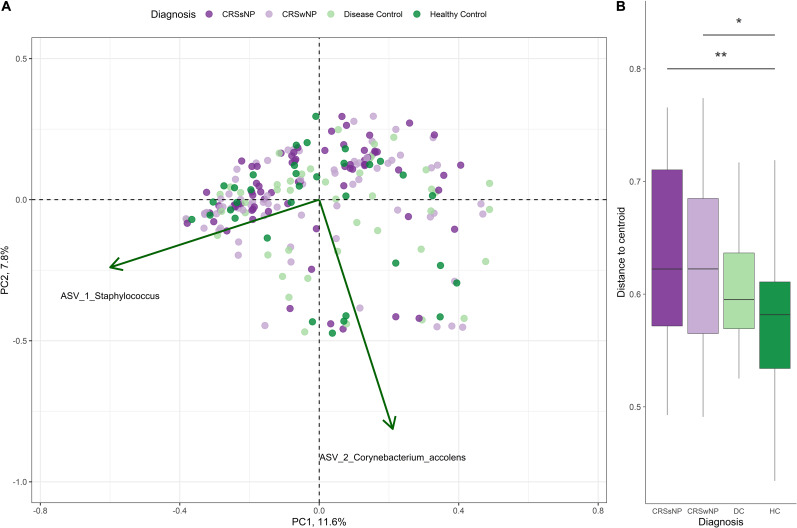
Principal coordinate analysis (PCoA) of sinus bacterial community structures in CRS and control patients. PCoA plots were constructed based on Bray-Curtis dissimilarity. **(A)** A two-dimensional PCoA plot with vectors indicating the correlation of important ASVs (green arrows) with community structure. **(B)** Box plot indicating the distances of samples to the centroid for CRS and control groups. Asterisks indicate that the multivariate homogeneity of group dispersions with Tukey’s honest significant difference resulted in a significant difference.

*Corynebacterium* and *Staphylococcus* were the most dominant bacterial genera across the dataset, accounting for 28.4 ± 25.7% (mean ± SD) and 17.7 ± 22.4% of genus-level assigned sequences, respectively. High inter-subject variation was observed for all four groups (Figure 3A). The number of observed ASVs was significantly higher in healthy subjects when compared to CRS (*p* = 0.0042 for both CRSwNP and CRSsNP) and disease control patients (*p* = 0.0178, Figure 3B). No significant differences were found when examining other alpha diversity metrics (Figures 3C,D). Bacterial community dispersion (indicating the level of microbiota variation among members of a given cohort) was significantly higher in CRS patients compared with healthy subjects, but not when compared with disease control patients (Figure 4). Two ASVs assigned to the most dominant genera *Staphylococcus* (ASV1) and *Corynebacterium* (ASV2) were identified as important drivers of overall bacterial community dispersion (Pearson’s *r* = −0.61, *p* < 0.001, and *r* = −0.80, *p* < 0.001, respectively).

### Effect of Disease State and Antibiotic Treatment on the Sinus Microbiota

PERMANOVA analyses investigated the extent of variation in the microbiota that could be attributed to variables such as age, asthma, disease state and study (Supplementary Table 3). Disease state accounted for 2.6% of the variability in the bacterial communities (*p* = 0.006) while study accounted for 4.1% (*p* = 0.02) in the final statistical model (Supplementary Table 4). However, PCoA analysis based on Bray-Curtis dissimilarity did not reveal an obvious clustering of samples based on study (data not shown). The effect of antibiotic treatment was evaluated according to several different variables: antibiotic prescription 12 months or 4 weeks prior to sample collection, number of antibiotic tablets and number of prescribed courses. Antibiotic prescription did not account for significant variation in the model, either independently or in conjunction with disease state (Supplementary Table 4).

**FIGURE 5:**
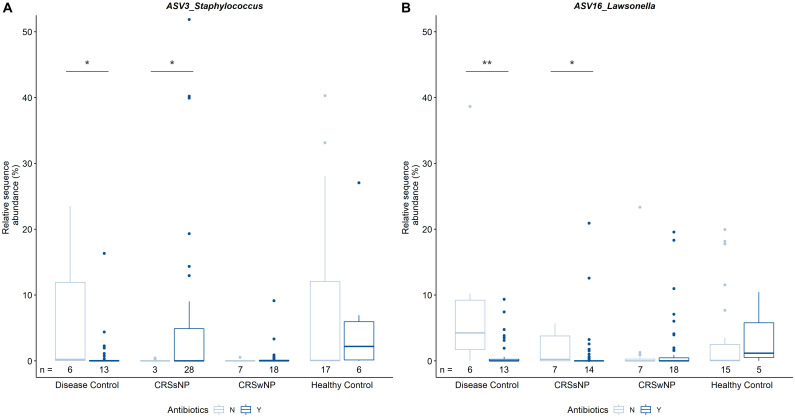
Pairwise comparisons of relative sequence abundances between CRS and control groups. Results for ASV3_Staphylococcus **(A)** and ASV16_Lawsonella **(B)** are shown. Of note, median relative sequence abundance in some subgroups approaches zero due to relatively low prevalence of ASVs 3 and 16 in those groups and *y*-axis scaling. The number of patients colonized by the respective ASV per group (n) is indicated below each bar. Statistical differences are indicated according to the following significance levels: *p < 0.05, **p < 0.01.

In order to determine the cumulative effects of antibiotics, data were analyzed based on antibiotic treatment during 12 months prior to sample collection. There were no obvious differences in bacterial community composition between overall antibiotic users and non-users, nor when further delineating antibiotic users based on disease state. In addition, antibiotic usage did not seem to influence bacterial alpha or beta diversity within or across cohorts. Pairwise comparisons of individual ASVs revealed differences in the relative sequence abundances of *Staphylococcus* (ASV3) and *Lawsonella* (ASV16) within groups based on disease state and antibiotic intake (Figure 5). It is of note that the median relative sequence abundance in some groups approaches zero due to low prevalence of these two ASVs (3 and 16) in the dataset. *Staphylococcus* was significantly more abundant in CRSsNP patients that were prescribed antibiotics when compared with CRSsNP patients that did not receive antibiotics. In contrast, *Staphylococcus* was significantly less abundant amongst antibiotic users compared to non-users in the disease control group. The relative abundance of *Lawsonella* was increased in both CRSsNP and disease control subjects without antibiotics when compared to subjects within the same group that were prescribed antibiotics.

LEfSe analyses were applied to identify potential bacterial biomarkers of antibiotic users and non-users across all cohorts. Several biomarkers were identified for antibiotic users and non-users (Supplementary Figure 1). One ASV belonging to the genus *Lawsonella* (ASV 16) and *Haemophilus influenzae* (ASV 13) are two representatives of biomarkers for subjects that did not receive antibiotics.

Bacterial community profiles and diversity did not differ between subjects that were prescribed antibiotics within 4 weeks prior to sample collection (*n* = 14) compared to subjects who did not receive any antibiotics during that period (*n* = 214), regardless of disease state. LEfSe analysis indicated an association of ASV3 (*Staphylococcus*) with subjects that did not receive antibiotics immediately prior to sample collection, independent of disease status. ASV32 (*Haemophilus*) may, however, be a potential biomarker for antibiotic users (Supplementary Figure 2).

## Discussion

The results from this study show that CRSsNP, CRSwNP, and disease control patients are prescribed a large amount of antibiotics in the year before their surgical procedures. This reflects the widespread prescription of antibiotics for the treatment of CRS as described by existing studies (Akkerman et al., 2005; Dubin et al., 2007; Smith et al., 2013; Gulliford et al., 2014; Schwartz et al., 2016). The number of prescribed antibiotics in this study is likely to be an under-estimation due to some missing data, and it is important to note that we are relying on prescription information to reflect patients’ actual antibiotic intake. One recent study has shown that despite intensive antibiotic prescription, no effects of antibiotic treatment on radiological (Lund-Mackay) and outcome scores (SNOT-22) were observed (Parasher et al., 2019). Our results indicated no differences in patient outcome between patients with pre-operative prescriptions of antibiotics and non-antibiotic users.

The data presented here revealed unexpectedly high antibiotic prescription rates in the disease control cohort that were equal to that in CRS patients. Unfortunately, the New Zealand electronic patient record system does not record the indication for each antibiotic prescription, which may have helped explain this observation. The high numbers of tablets and courses prescribed in the disease control group are concerning, as the administration of antibiotics is generally associated with a variety of side-effects. Negative effects related to antibiotic usage range from mild (e.g., gastrointestinal) to more severe (e.g., cardiovascular disease) (Cheng et al., 2015; Winkel et al., 2015; Walker et al., 2019).

### Healthy Controls but Not Disease Controls Show a Distinct Bacterial Community From CRS Patients

This study reaffirms previous observations that *Staphylococcus* and *Corynebacterium* are dominant sinonasal taxa (Biswas et al., 2015; Hoggard et al., 2017a; Wagner Mackenzie et al., 2017; Chalermwatanachai et al., 2018; Paramasivan et al., 2019; Rom et al., 2019). Our results show significant differences in bacterial alpha and beta diversities only between CRS subgroups and the healthy group, but not between CRS and disease control patients. Other studies have found no differences between CRS and disease control patients (Paramasivan et al., 2019). Antibiotic treatment may reduce bacterial diversity (Liu et al., 2013), so the lack of significant difference in richness and diversity between CRS and disease control patients in this study could be due to the high antibiotic use in the disease control group. Surprisingly, however, no differences in bacterial profiles were observed between antibiotic users and non-users in our study. This suggests that observed decreases in diversity are not mediated by antibiotics alone but rather by a combination of medical treatment and health status. This dynamic relationship between disease, treatment and microbial community has been previously described in the human gastrointestinal tract (Shreiner et al., 2015).

Studies typically use a control cohort with patients that undergo sinus surgery for reasons unrelated to CRS. However, our findings suggest that those patients may not represent an ideal control and highlight the importance of a healthy control group that accurately represents a normal sinus microbial community. The lack of truly healthy controls in many studies may contribute to the paucity of evidence in the current literature for a distinct CRS-associated microbiome.

### Cumulative and Immediate Antibiotic Treatment Effects on the Sinus Bacterial Community Are Minimal and Unpredictable

While no community-wide effects were observed, two ASVs were differentially abundant based on antibiotic intake within groups. *Lawsonella* is prevalent in human sinuses (Escapa et al., 2018) and in this dataset was associated with healthy subjects. The abundance of *Lawsonella* (ASV16) was decreased in CRSsNP and disease control patients prescribed antibiotics. This result highlights the susceptibility of this bacterium to antibiotics such as doxycycline (Sturm et al., 2019) and demonstrates the potentially deleterious unintended effects of long-term antibiotic treatment on health-associated microbes.

In-depth analyses of the immediate effects of antibiotic treatment within groups were limited due to small sample sizes. When comparing cumulative and immediate effects of antibiotics on bacterial biomarkers, however, our findings further suggest that different ASVs belonging to the same genera (i.e., *Haemophilus*) can be found as biomarkers for both antibiotic usage and no usage. This unpredictability could be due to differences in antibiotic resistance patterns and microbial community dynamics.

### Significance and Study Limitations

Detecting effects of antibiotic treatment on the sinus microbiome is challenging. It is likely that potential effects are – like the sinus microbiota in general – highly individual (Liu et al., 2013; Wagner Mackenzie et al., 2019b), rather than manifesting as clear patterns across entire cohorts. The analysis tools that are presently used to measure and compare 16S rRNA gene-based microbial community data may be insufficient to detect such subtle differences.

This study evaluated the association of disease state and treatment with the sinonasal bacterial community. While most of the sequencing data used in this study were generated in previously published research, there are some limitations that should be considered when drawing conclusions. A major challenge lies in quantifying the extent of antibiotic treatment. Many studies use patient questionnaires to estimate the number of days on which antibiotics were used throughout a certain time period. Here, we used an electronic medical database to directly extract information on the absolute amount of antibiotics prescribed per patient. This allowed us to eliminate recall bias in CRS patients. We obtained data on type and amount of antibiotics as well as the order in which they were prescribed, however, there is no information on the timeline and indication for antibiotic treatment within the one year prior to surgery. Not knowing if antibiotics were prescribed for exacerbation of CRS or other reasons further limits our interpretation of their impact on clinical and microbiological outcomes. Antibiotic prescription rates are not uniform and the time between consecutive prescriptions can vary significantly between patients. However, antibiotic data immediately preceding sample collection exist and were analyzed.

Many CRS studies published to date analyze sequencing data by clustering sequences into operational taxonomic units (OTUs) at 97% sequence similarity. OTUs are study-specific and cannot be compared across different datasets. For this study, we used a recently developed pipeline which groups sequences according to amplicon sequence variants (ASVs). While a consensus has not yet been reached on the best way to analyze amplicon data, one advantage of employing ASVs is that these sequence entities are comparable between datasets. Differences in bioinformatic pipelines between this work and other studies could explain some of the observed discrepancies.

All analyses in the current study were conducted based on cross-sectional data. This is generally problematic when trying to unravel differences in microbial community profiles, particularly in a setting where subjects naturally show high inter-subject variability. Although relatively high numbers of samples were analyzed, observing significant and large differences between groups remains challenging. Without a longitudinal study design, the question remains as to what role continuous antibiotic prescriptions play in the breakdown of the bacterial community. Shifts in microbial community composition due to antibiotic exposure can last for several months before the microbiome recovers (Jernberg et al., 2010; Wagner Mackenzie et al., 2019b). More effective treatment strategies for CRS are required so that patients are not unnecessarily exposed to the risks of antibiotics, including the development of resistance.

## Data Availability Statement

The datasets presented in this study can be found in the NCBI Sequence Read Archive. The accession number(s) can be found below: Supplementary Table 1, PRJNA639382 (healthy controls).

## Ethics Statement

The studies involving human participants were reviewed and approved by New Zealand Health and Disability Ethics Committee (NTX/08/12/126). The patients/participants provided their written informed consent to participate in this study.

## Author Contributions

CL, KB, RD, and MT planned and conceived the study. CL carried out the experiments, the data analysis and took the lead in writing the manuscript with support from KB, RD, MT, and BW. BW also assisted with the data analysis. JJ and MZ helped to collect patients’ demographic and clinical information. All authors contributed to the article and approved the submitted version.

## Conflict of Interest

The authors declare that the research was conducted in the absence of any commercial or financial relationships that could be construed as a potential conflict of interest.

## Supplementary Material

Supplementary Figure 1 LEfSe analysis for antibiotic users (Y) and non-users (N) across all cohorts. Differential feature plots for ASV_16 and ASV_13 are shown.

Supplementary Figure 2 LEfSe analysis for all subjects that received (Y) or did not receive (N) any antibiotic in the month prior to sample collection. Differential feature plots for ASV_3 and ASV_32 are shown.

Supplementary Table 1 Summary of studies included in the meta-analysis.

Supplementary Table 2 *p*-values for pairwise comparisons for significant variables.

Supplementary Table 3 PERMANOVA results for independent variable testing. Partitioning of variance is reflected in the R^2^ value. *p*-values were obtained using 999 permutations of levels within each factor.

Supplementary Table 4 PERMANOVA results for control and testvariables combined in the statistical model. Only those variables that were identified as significant independently (in Supplementary Table 3) were retained in this final model. Partitioning of variance is reflected in the R^2^ value. *p*-values were obtained using 999 permutations of levels within each factor.
