# Supplementary figures and images for: Antibiotic Treatment for Chronic Rhinosinusitis: Prescription Patterns and Associations With Patient Outcome and the Sinus Microbiota
Source: Front Microbiol. 2020 Dec 22;11:595555. doi: 10.3389/fmicb.2020.595555 (PMC7782326; doi:10.3389/fmicb.2020.595555)

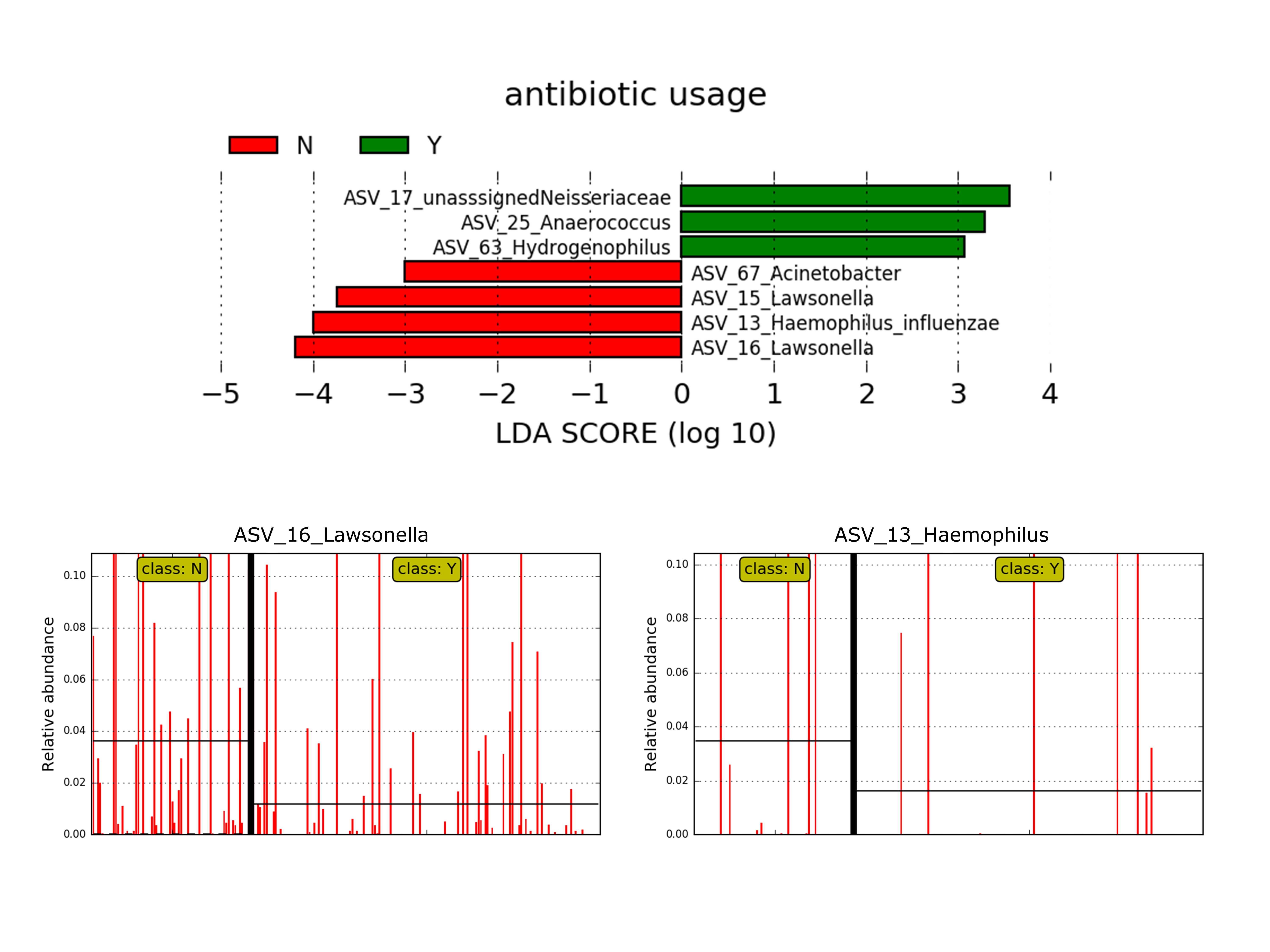

Supplement: Supplementary Figure 1 — LEfSe analysis for antibiotic users (Y) and non-users (N) across all cohorts. Differential feature plots for ASV_16 and ASV_13 are shown. [file Image_1.tif]

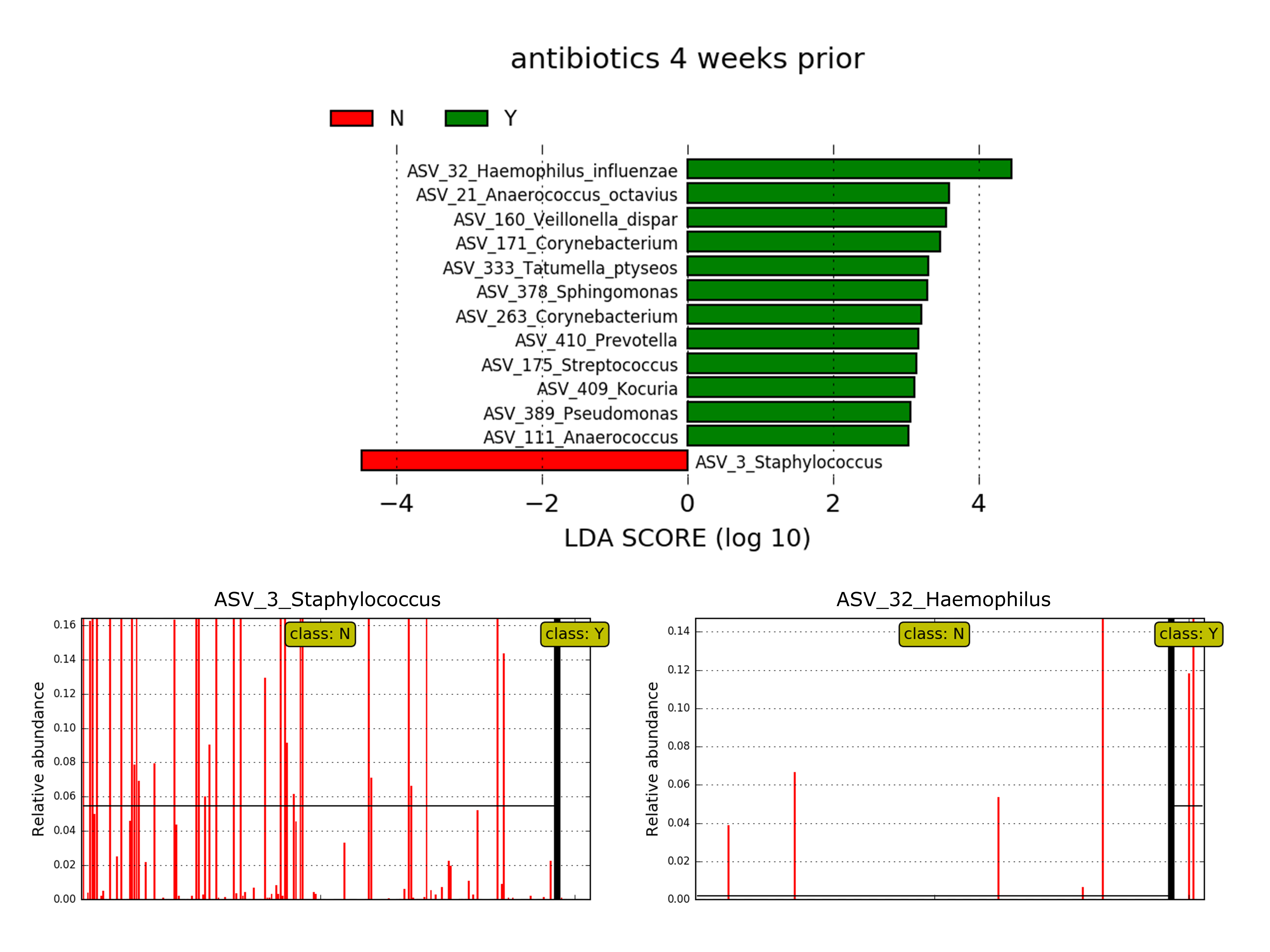

Supplement: Supplementary Figure 2 — LEfSe analysis for all subjects that received (Y) or did not receive (N) any antibiotic in the month prior to sample collection. Differential feature plots for ASV_3 and ASV_32 are shown. [file Image_2.tif]
