# Supplementary material for: Antibiotic Treatment for Chronic Rhinosinusitis: Prescription Patterns and Associations With Patient Outcome and the Sinus Microbiota
Source: Front Microbiol. 2020 Dec 22;11:595555. doi: 10.3389/fmicb.2020.595555 (PMC7782326; doi:10.3389/fmicb.2020.595555)
Supplement: Supplementary Table 1 — Summary of studies included in the meta-analysis. [file Data_Sheet_1.docx]

*Table S1. Summary of studies included in the meta-analysis*

| Study | Subjects (n) | Sample site | Sample type | Sequencing platform | NCBI data accession |
| --- | --- | --- | --- | --- | --- |
| Jain et al., 2018 | 24 | left middle meatus | paired swabs | Illumina MiSeq 2 × 300 bp, paired-end | NA |
| Wagner Mackenzie et al., 2019 | 54 | left middle meatus | paired swabs | Illumina MiSeq 2 × 300 bp, paired-end | PRJNA512906 |
| Lux et al., 2020 | 18 | left middle meatus | paired swabs | Illumina MiSeq 2 × 300 bp, paired-end | PRJNA638969 |
| Hoggard et al., 2017 | 76 | left middle meatus | paired swabs | Illumina MiSeq 2 x 250 and 2 × 300 bp, paired-end | SRP092370 |
| Biswas et al., 2017 | 19 | left middle meatus | paired swabs | Illumina MiSeq 2 × 300 bp, paired-end | PRJNA390854 |
| Biswas et al., 2019 | 10 | ethmoidal sinus | tissue biopsies | Illumina MiSeq 2 × 300 bp, paired-end | PRJNA482256 |

Table S2. p-values for pairwise comparisons for significant variables

| Variables^a^ | CRSsNP vs CRSwNP | CRSsNP vs Disease Control | CRSsNP vs Healthy Control | CRSwNP vs Disease Control | CRSwNP vs Healthy Control | Disease Control vs Healthy Control |
| --- | --- | --- | --- | --- | --- | --- |
| Age | 0.384 | 0.894 | **< 0.001** | 0.918 | **< 0.001** | **< 0.001** |
| Gender | 0.163 | 0.229 | 0.878 | **0.007** | 0.328 | 0.229 |
| Ethnicity^b^ | 0.646 | 0.753 | **< 0.001** | 0.442 | **< 0.001** | **0.007** |
| Asthma | **0.015** | **0.003** | NA | **< 0.001** | NA | NA |
| Antibiotic usage | 0.223 | 0.633 | **< 0.001** | 0.183 | **< 0.001** | **< 0.001** |
| Antibiotic usage (1 month prior to sample collection) | 0.15 | 0.3 | NA | **0.022** | NA | NA |
| Number of antibiotics (tablets) | **0.013** | 0.1454 | NA | 0.187 | NA | NA |
| Lund-Mackay score | **< 0.001** | **< 0.001** | NA | **< 0.001** | NA | NA |
| SNOT-22 score | 0.769 | **< 0.001** | NA | **< 0.001** | NA | NA |

^a^Categorical variables were analysed using pairwise *Chi* squared test or Fisher’s exact test (for groups with n<5) and continuous variables were tested by computing Tukey Honest Significant Difference or Dunn’s test with the Benjamini-Hochberg adjustment for multiple comparison. Significant p values (p < 0.05) are noted in bold. ^b^European vs “Other”

Table S3. PERMANOVA results for independent variable testing. Partitioning of variance is reflected in the R^2^ value. p-values were obtained using 999 permutations of levels within each factor.

| Variable | R^2^ | *p*-value |
| --- | --- | --- |
| Study | **0.047** | **0.002** |
| Disease state | **0.021** | **0.002** |
| Ethnicity | 0.028 | n.s. |
| Gender | 0.006 | 0.069 |
| Age | **0.010** | **0.001** |
| Smoker | 0.008 | n.s. |
| Polyposis | 0.005 | n.s. |
| Asthma | **0.012** | **0.001** |

Table S4. PERMANOVA results for control and test variables combined in the statistical model. Only those variables that were identified as significant independently (in Table S3) were retained in this final model. Partitioning of variance is reflected in the R^2^ value. p-values were obtained using 999 permutations of levels within each factor.

| Variable | R^2^ | *p*-value |
| --- | --- | --- |
| Study | **0.041** | **0.020** |
| Disease state | **0.026** | **0.006** |
| Asthma | 0.009 | **0.013** |
| Age | - | n.s |
| Antibiotics (yes/no 12 months) | - | n.s. |
| Antibiotics (yes/no 4 weeks) | - | n.s. |
| Number of antibiotic tablets | - | n.s. |
| Number of antibiotic courses | - | n.s. |
| Lund-Mackay score | 0.013 | **0.015** |
| SNOT-22 score | - | n.s. |
